# Supplementary material for: Combination of acalabrutinib with lenalidomide and rituximab in relapsed/refractory aggressive B-cell non-Hodgkin lymphoma: a single-arm phase II trial
Source: Nat Commun. 2024 Mar 30;15:2776. doi: 10.1038/s41467-024-47198-4 (PMC10981676; doi:10.1038/s41467-024-47198-4)
Supplement: Supplementary file 1 — Supplementary Information [file 41467_2024_47198_MOESM1_ESM.pdf]

SUPPLEMENTARY FIGURES

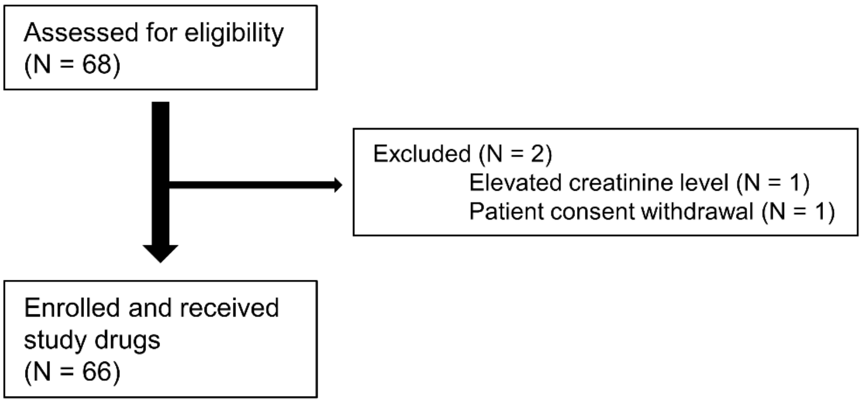

Supplementary Figure 1. CONSORT diagram for this study

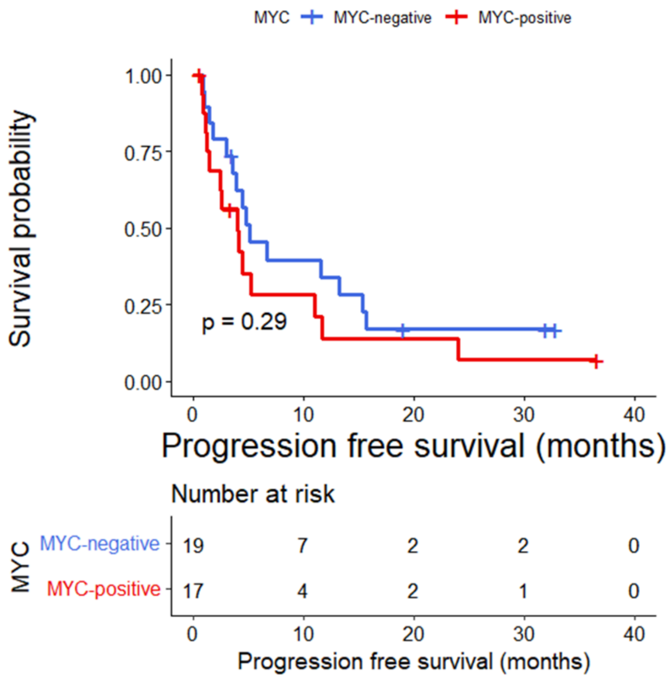

Supplementary Figure 2. Progression free survival of Bcl-2 positive patients according to Myc IHC status

The Kaplan-Meier curves show PFS according to Myc IHC status among Bcl-2 positive patients. The red line represents Myc positive group, and the blue line represents the Myc negative status group. Cross marks on the curves depict censored data. Abbreviations: IHC, immunohistochemistryse; PFS, progression free survival

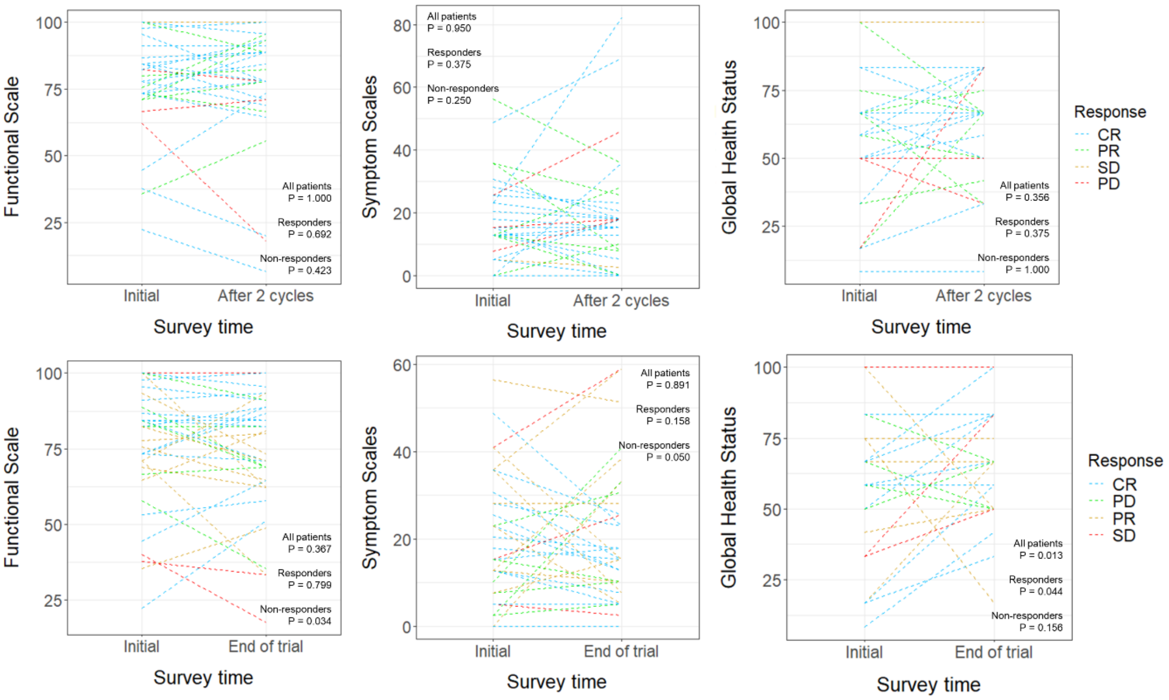

Supplementary Figure 3. Quality of life assessments by EORTC-QLQ C30

Each line represents changes in each patient. The colors of the lines represent the responses. The p-values for Wilcoxon signed-rank test are shown in each plot.

Supplementary Figure 4

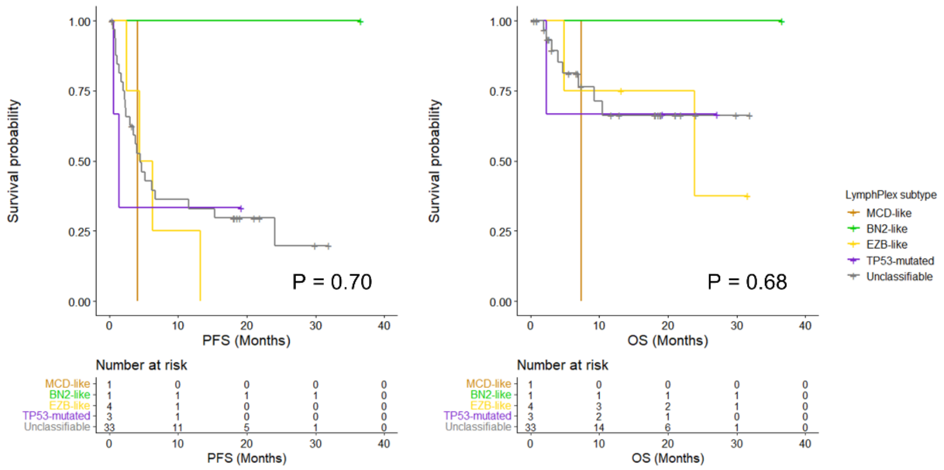

Supplementary Figure 4. Survival of patients according to LymphPlex classification subtype

The Kaplan-Meier curves show PFS and OS according to LymphPlex classification subtypes. Cross marks on the curves depict censored data. Abbreviations: OS, overall survival; PFS, progression-free survivalProgression free survival of patients according to BTK group

The Kaplan-Meier curves show PFS according to BTK IHC group by single-molecule fluorescence imaging. The red line represents BTK high group, and the blue line represents BTK low group. Cross marks on the curves depict censored data. Abbreviations: BTK, Bruton's tyrosine kinase; PFS, progression free survival

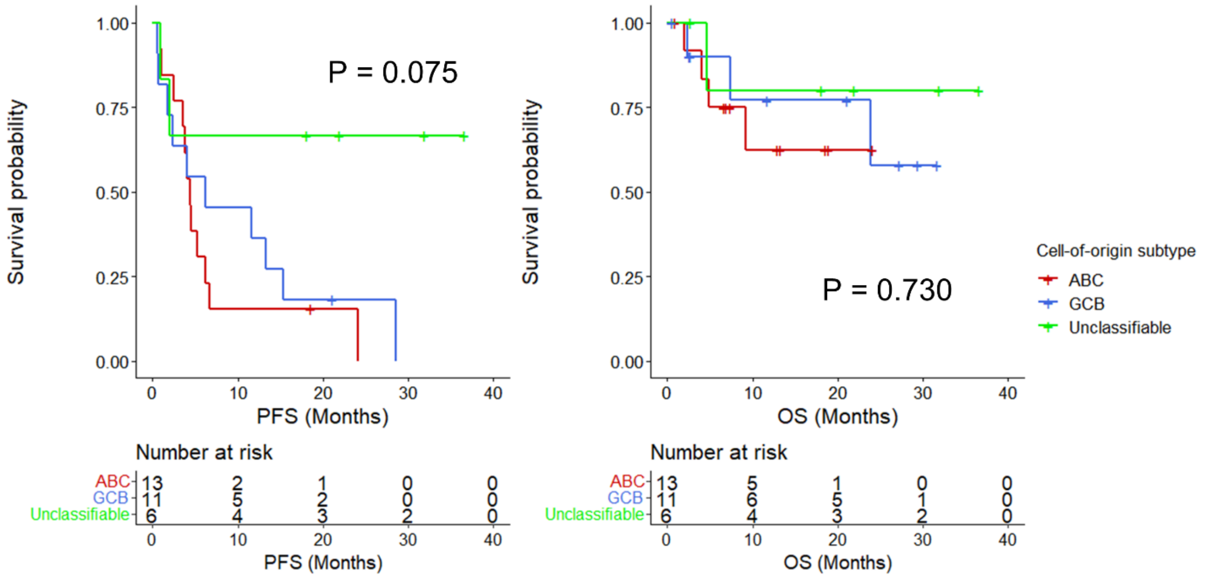

Supplementary Figure 5. Survival of patients according to cell-of-origin subtype

The Kaplan-Meier curves show PFS and OS according to cell-of-origin subtype defined by expression profiling through RNA-sequencing. Cross marks on the curves depict censored data. Abbreviations: ABC, activated B-cell subtype; GCB, germinal center B-cell subtype; OS, overall survival; PFS, progression-free survival

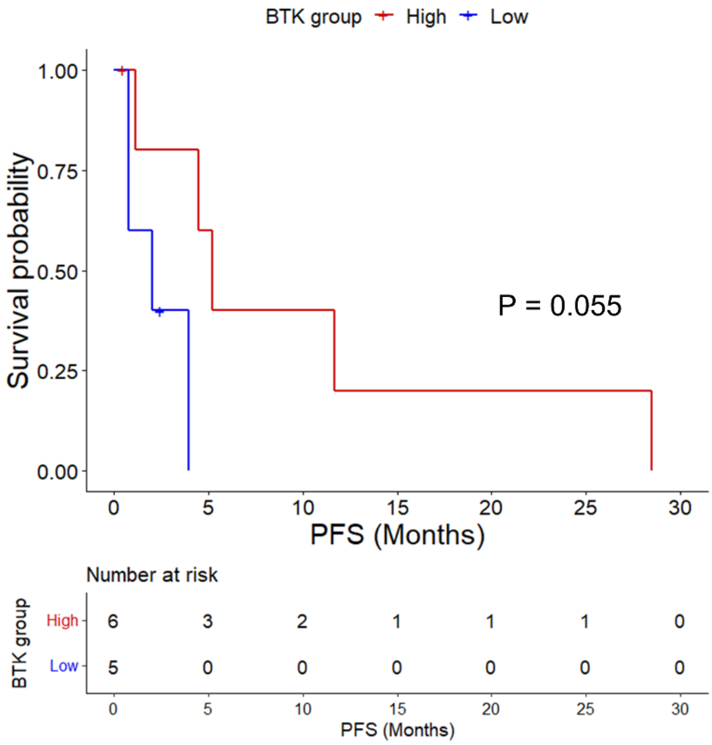

Supplementary Figure 6. Progression free survival of patients according to BTK group

The Kaplan-Meier curves show PFS according to BTK IHC group by single-molecule fluorescence imaging. The red line represents BTK high group, and the blue line represents BTK low group. Cross marks on the curves depict censored data. Abbreviations: BTK, Bruton's tyrosine kinase; IHC, immunohistochemistry; PFS, progression free survival

SUPPLEMENTARY TABLES

Supplementary Table 1. Target genes for DNA sequencing (258 genes)

	1	2	3	4	5	6	7	8	9	10
1	ABL1	ACTB	AKT1	AKT2	AKT3	ALK	APC	AR	ARHGEF1	ARID1A
2	BCL6	BCL7A	BCOR	BIRC3	BRAF	BRCA1	BRCA2	BRIP1	BTG1	BTK
3	CD79A	CD79B	CD83	CDH1	CDK12	CDK4	CDK6	CDKN2A	CEBPA	CHD2
4	DDX3X	DNMT3A	EGFR	EGR2	EIF2AK3	ELF4	EP300	EPHA7	ERBB2	ERBB3
5	FANCL	FAS	FBXW7	FGF1	FGF10	FGF14	FGF2	FGF23	FGF3	FGF4
6	FLT3	FOXL2	FOXO1	FYN	GEN1	GNA11	GNA13	GNAI2	GNAQ	GNAS
7	HLA-B	HNF1A	HRAS	HVCN1	IDH1	IDH2	IGF1R	IKZF1	IL10RA	IL6
8	KMT2A	KMT2D	KRAS	LTB	LYN	MAP2K1	MAP2K2	MCL1	MDM2	MDM4
9	MSH3	MSH6	MTOR	MUTYH	MYC	MYCL	MYCN	MYD88	NBN	NF1
10	NTRK1	OSBPL10	P2RY8	PALB2	PDCD1LG2	PDE4DIP	PDGFRA	PDGFRB	PIK3CA	PIK3CB
11	PRDM1	PRKCB	PTCH1	PTEN	PTPN1	PTPN11	PTPN6	PTPRD	RAD51	RAD51B
12	RPS15	RPS6KB1	RUNX1	S1PR2	SETBP1	SETD2	SF3B1	SGK1	SLX4	SMAD4
13	SYK	TERT	TET2	TLR2	TMEM30A	TNFAIP3	TNFRSF14	TOX	TP53	TSC1
14	ASXL1	ATM	ATR	AURKA	B2M	BAK1	BAP1	BARD1	BCL10	BCL2
15	CALR	CARD11	CCND1	CCND2	CCND3	CCNE1	CCR7	CD274	CD58	CD70
16	CHEK1	CHEK2	CIITA	CREBBP	CSF1R	CSF3R	CTNNB1	CXCR4	DAZAP1	DDR2
17	ERBB4	ERCC1	ERCC2	ERG	ESR1	ETS1	ETV6	EZH2	FAM175A	FANCI
18	FGF5	FGF6	FGF7	FGF8	FGF9	FGFR1	FGFR2	FGFR3	FGFR4	FLT1
19	GRHPR	HIST1H1B	HIST1H1C	HIST1H1D	HIST1H1E	HIST1H2AC	HIST1H2AM	HIST1H2BC	HIST1H2BK	HLA-A
20	IL6R	INPP4B	IRF4	IRF8	ITPKB	JAK2	JAK3	KDR	KIT	KLF2
21	MED12	MEF2B	MET	MIR17HG	MLH1	MLLT3	MPEG1	MPL	MRE11A	MSH2
22	NFKB2	NFKBIA	NFKBIE	NFKBIZ	NOTCH1	NOTCH2	NOTCH3	NPM1	NRAS	NRG1
23	PIK3CD	PIK3CG	PIK3R1	PIM1	PIM2	PLCG2	PMS2	POLG	POT1	PPP2R2A
24	RAD51C	RAD51D	RAD54L	RARA	RB1	REL	RET	RHOA	RICTOR	ROS1
25	SMARCB1	SMO	SOCS1	SPEN	SPIB	SRC	SRSF2	STAT3	STAT6	STK11
26	TSC2	UBE2A	VHL	XBP1	XPO1	XRCC2	ZC3H12A	ZFP36L1		

Supplementary Table 2. Target exons for RNA sequencing

Genes	Target regions
BCL10	Whole exons
BCL2	Whole exons
BCL6	Whole exons
CBFB	Whole exons
CCND1	Whole exons
CCND3	Whole exons
CD274	Whole exons
DUSP22	Whole exons
ETV6	Whole exons
ITK	Whole exons
MAF	Whole exons
MALT1	Whole exons
MYH11	Whole exons
PBX1	Whole exons
PDCD1LG2	Whole exons
RARA	Whole exons
RUNX1	Whole exons
RUNX1T1	Whole exons
SYK	Whole exons
TP63	Whole exons
IGHA2_1	Target exon
IGHE_3	Target exon
IGHG4_1	Target exon
IGHG2_1	Target exon
IGHA1_1	Target exon
IGHG1_9	Target exon
IGHG3_1	Target exon
IGHD_1	Target exon
IGHM_1	Target exon
IGHJ6_1	Target exon
IGHJ5_1	Target exon
IGHJ4_1	Target exon
IGHJ3_1	Target exon
IGHJ2_1	Target exon
IGHJ1_1	Target exon
IGHD7-27_1	Target exon
IGHD1-26_1	Target exon
IGHD6-25_1	Target exon
IGHD5-24_1	Target exon
IGHD4-23_1	Target exon
IGHD3-22_1	Target exon

IGHD2-21_1	Target exon
IGHD1-20_1	Target exon
IGHD6-19_1	Target exon
IGHD5-18_1	Target exon
IGHD4-17_1	Target exon
IGHD3-16_1	Target exon
IGHD2-15_1	Target exon
IGHD1-14_1	Target exon
IGHD6-13_1	Target exon
IGHD5-12_1	Target exon
IGHD4-11_1	Target exon
IGHD3-10_1	Target exon
IGHD3-9_1	Target exon
IGHD2-8_1	Target exon
IGHD1-7_1	Target exon
IGHD6-6_1	Target exon
IGHD5-5_1	Target exon
IGHD4-4_1	Target exon
IGHD3-3_1	Target exon
IGHD2-2_1	Target exon
IGHD1-1_1	Target exon
IGHV6-1_1	Target exon
IGHV1-2_1	Target exon
IGHV1-3_1	Target exon
IGHV4-4_1	Target exon
IGHV2-5_1	Target exon
IGHV3-7_1	Target exon
IGHV1-8_1	Target exon
IGHV3-9_1	Target exon
IGHV3-11_1	Target exon
IGHV3-13_1	Target exon
IGHV3-15_1	Target exon
IGHV3-16_1	Target exon
IGHV1-18_1	Target exon
IGHV3-20_1	Target exon
IGHV3-21_1	Target exon
IGHV3-23_1	Target exon
IGHV1-24_1	Target exon
IGHV2-26_1	Target exon
IGHV4-28_1	Target exon
IGHV3-30_1	Target exon
IGHV4-31_1	Target exon
IGHV3-33_1	Target exon

IGHV4-34_1	Target exon
IGHV3-35_1	Target exon
IGHV3-38_1	Target exon
IGHV4-39_1	Target exon
IGHV3-43_1	Target exon
IGHV1-45_1	Target exon
IGHV1-46_1	Target exon
IGHV3-48_1	Target exon
IGHV3-49_1	Target exon
IGHV5-51_1	Target exon
IGHV3-53_1	Target exon
IGHV1-58_1	Target exon
IGHV4-59_2	Target exon
IGHV4-61_1	Target exon
IGHV3-64_1	Target exon
IGHV3-66_1	Target exon
IGHV1-69_1	Target exon
IGHV2-70_1	Target exon
IGHV3-72_1	Target exon
IGHV3-73_1	Target exon
IGHV3-74_1	Target exon
IGHV7-81_1	Target exon

Supplementary Table 3. The list of antibodies used for immunohistochemistry in this study

	Antibodies	Cat. No.	Clone	Company	Dilution	Antigen retrieval	Endogenous peroxidase blocking	Incubation time with primary antibody	Secondary and chromogen	Machine
1	Btk	#8547	D3H5	Cell Signaling Technology	1:100	20 min with ER2 Buffer (pH 8.0) in 97 ℃ Bond-Rx	5min	30min with Bond-max autoimmunostainer (Leica Biosystem, Melbourne, Australia)	10 min Bond-max autoimmunostainer (Leica Biosystem, Melbourne, Australia) using Bond™ Polymer refine detection, DS9800 (Vision Biosystems, Melbourne, Australia)	Bond-max autoimmunostainer (Leica Biosystem)
2	Btk	#56044	D6T2C	Cell Signaling Technology	1:100	20 min with ER2 Buffer (pH 8.0) in 97 ℃ Bond-Rx	5min	30min with Bond-max autoimmunostainer (Leica Biosystem, Melbourne, Australia)	10 min Bond-max autoimmunostainer (Leica Biosystem, Melbourne, Australia) using Bond™ Polymer refine detection, DS9800 (Vision Biosystems, Melbourne, Australia)	Bond-max autoimmunostainer (Leica Biosystem)

Supplementary Note 1. Clinical trial protocol

STUDY SYNOPSIS

**Study Title:**	Phase II trial of acalabrutinib with rituximab and lenalidomide in relapsed/refractory B-cell non-Hodgkin's lymphoma
**Primary Investigator:**	Youngil Koh, Seoul National University Hospital
**Purpose:**	We plan to evaluate the efficacy of the combination regimen of acalabrutinib with rituximab and lenalidomide in patients with relapsed/refractory B-cell non-Hodgkin's lymphoma, and eventually improve the prognosis of these patients.
**Study Design:**	Prospective, open-label, multicenter, phase II clinical trial
**Study Drug:**	ACALABRUTINIB<br>RITUXIMAB<br>LENALIDOMIDE
**Comparator:**	Not applicable
**Study Objectives:**	Primary Objective(s):<br>▪ Overall response rate by Lugano criteria<br>Secondary Objective(s):<br>▪ DOR<br>▪ CR rate<br>▪ PFS<br>▪ OS<br>▪ Treatment-emergent AE, SAE, AEs leading to discontinuation of investigational medicinal products<br>Exploratory Objective(s):<br>▪ Next Generation Sequencing study with pretreatment biopsy of tumor
**Study Design:**	Prospective open-label phase II study, single-arm
**Efficacy and Safety Parameters:**	Efficacy parameters: Lugano criteria<br>Safety parameters: NCI-CTCAE version 4.03
**Sample Size:**	66 (single-arm study)

**Dose Regimen/Route of Administration:**	Acalabrutinib is provided as hard gelatin capsules for oral administration. Acalabrutinib 100 mg will be administered approximately every 12 hours from day 1 to day 28.<br><br>Rituximab is provided as single-use vials for intravenous administration only. Rituximab 375 mg/m^2 will be administered on day 1.<br><br>Lenalidomide is provided as opaque hard capsules for oral administration. Lenalidomide 20 mg will be administered once daily from day 1 to day 21.<br><br>Day 1 to day 28 is considered one cycle, and cycles will be repeated every 4-weeks, for 6 cycles.<br><br>After 6 cycles, maintenance of acalabrutinib 100mg bid will be administered up to 1 year for subjects whose best responses- to the regimen are partial response or complete remission.

ABBREVIATIONS

AE	adverse event
ALT	alanine aminotransferase
ANC	absolute neutrophil count
AST	aspartate aminotransferase
BID	twice per day (dosing)
BOR	best overall response
BTK	Bruton tyrosine kinase
BUN	blood urea nitrogen
CBC	complete blood count
CIs	confidence intervals
CLL	chronic lymphocytic leukemia
CLcr	Creatinine clearance
CR	complete response (remission)
CRF	case report form
CT	computed tomography
CTCAE	Common Terminology Criteria For Adverse Events
CYP	cytochrome p450
DOR	duration of response
ECG	Electrocardiogram
ECOG	Eastern Cooperative Oncology Group
FDA	Food and Drug Administration
GCB	germinal center B cell
HBV	hepatitis B virus
hCG	Human chorionic gondaotropin
HCV	hepatitis C virus
HIV	human immunodeficiency virus
ICF	informed consent form
IRB	institutional review board
IV	intravenous or intravenously

LDH	lactate dehydrogenase
MRI	Magnetic resonance imaging
NHL	non-Hodgkin’s lymphoma
ORR	objective response rate
OS	overall survival
PCR	Polymerase chain reaction
PFS	progression-free survival
PD	progressive disease
PR	partial response (remission)
QD	once per day (dosing)
R/R	relapsed/refractory
SAE	serious adverse event
SD	stable disease
SFU	safety follow-up
ULN	upper limit of normal

PROTOCOL

1. Study Title

Phase II trial of acalabrutinib with rituximab and lenalidomide in relapsed/refractory B-cell non-Hodgkin's lymphoma

2. Study Centers

Subjects will be enrolled in approximately 10 centers in South Korea. Center accrual would be done through CISL (http://www.cisl.co.kr)

3. Primary Investigator

Youngil Koh, Seoul National University Hospital

4. Provision of Medication

Acalabrutinib: AstraZeneca

Rituximab: Celltrion

Lenalidomide: Samyang Holdings

5. Estimated Study Period

From IRB approval to November 30th, 2024 (6 years)

6. Subject Disease

Relapsed/refractory B-cell non-Hodgkin's lymphoma (R/R B-cell NHL)

7. Background

Since the era of Rituximab, treatment for CD20 positive aggressive B cell non-Hodgkin's lymphoma(NHL) has significantly advanced, achieving complete remission as high as 76%.[1,2] However, as much as one third of patients experience relapse of devastating disease.[3] There are certain proportion of patients primarily refractory to commonly used first line rituximab-based regimens.[4] These relapsed/refractory aggressive B-NHL have poor prognosis, and there is no consensus salvage regimen for such diseases.[5] High-dose chemotherapy with stem cell rescue is unsatisfactory.[6] Moreover, many fragile patients are unfit for salvage cytotoxic chemotherapy and/or high-dose chemotherapy.[7]

Hence, most of patients with relapsed/refractory aggressive B-cell NHL is ultimately candidate for less-cytotoxic drugs with targeted approach. Among many newly developed agents, lenalidomide is one of the most promising agents in the field of B-cell NHL.[8] Recent trials showed the favorable efficacy with good tolerability of lenalidomide and rituximab combination in B-cell NHL.[9,10,11,12] However, these results are still not enough for aggressive B-cell NHL. Considering the dismal outcome of R/R aggressive B-NHL, further investigation is necessary to improve outcome of relapsed/refractory aggressive B-cell NHL.

Bruton tyrosine kinase (BTK) is a non-receptor enzyme in the Tec kinase family that is expressed among cells of hematopoietic origin, including B-cells, myeloid cells, mast cells, and platelets, where it regulates multiple cellular processes including proliferation, differentiation, apoptosis, and cell migration.[13,14,15] In addition, BTK-dependent activation of mast cells, myeloid cells, and other immunocytes in peritumoral inflammatory stroma has been shown to sustain the complex microenvironment needed for lymphoid and solid tumor maintenance.[16,17,18] Taken together, these findings suggest inhibition of BTK may offer an attractive strategy for treating B-cell neoplasms.

In fact, BTK inhibitor as single treatment showed promising efficacy in relapsed/refractory diffuse large B-cell lymphoma.[19] In a trial of ibrutinib for patients with relapsed/refractory mantle cell lymphoma, ORR of 68% and CR of 21%, median progression free survival of 13.9 months, with favorable toxicity profiles, suggesting potential for effective combination treatments.[20] In vitro data have shown potential of BTK inhibition to augment cytotoxic effect of lenalidomide.[21] BTK inhibitor with lenalidomide and rituximab combination showed ORR of 76% with 56% CR rate in previous clinical trial.[22] Hence, theoretically, combination of BTK inhibitor with rituximab and lenalidomide could be one of the ideal regimens against entire R/R B-cell NHL. Acalabrutinib is a potent inhibitor of BTK in vitro and in vivo, and acalabrutinib shows improved selectivity for BTK compared with ibrutinib, thus it can be expected to be potentially more effective and less toxic.

In the meantime, recent study on genomic classification using whole exome sequencing of diffuse large B-cell lymphoma (DLBCL) suggested that the response to BTK inhibitor may differ between DNA subtypes.[23] Therefore, prospective analysis on the biomarker for response to BTK inhibitor is necessary.

Here, we plan to evaluate the efficacy of the combination regimen of acalabrutinib with rituximab and lenalidomide in patients with relapsed/refractory B-cell non-Hodgkin's lymphoma and the biomarker for the regimen.

8. Information and Provision of Investigational Medicinal Products

(1) Acalabrutinib

The investigational product, acalabrutinib capsules for oral administration, is supplied as yellow and blue, opaque hard gelatin capsules, with 100 mg of acalabrutinib as the active ingredient. Each capsule also contains compendial inactive ingredients: silicified microcrystalline cellulose, which is composed of microcrystalline cellulose and colloidal silicon dioxide, partially pregelatinized starch, sodium starch glycolate, and magnesium stearate. The capsule shell contains gelatin, titanium dioxide, yellow iron oxide and indigotine (FD&C Blue 2).

Acalabrutinib will be provided in white, high-density polyethylene bottles.

(2) Rituximab (Truxima)

Rituximab vials (100mg/10mL and 500mg/50mL) are stable at 2℃-8℃ (36℉-46℉). Rituximab vials should be protected from direct sunlight. Rituximab vials should not be frozen or shaken.

(3) Lenalidomide (Lenalid)

Lenalidomide capsules (2.5mg, 5mg, 10mg and 20mg) for oral administration is supplied as opaque hard capsules. Lenalidomide should be stored in a closed container at room temperature,

away from heat, moisture, and direct light. Care should be exercised in the handling of lenalidomide. Lenalidomide capsules should not be opened or broken.

(4) Packaging and Labeling
The investigational medicinal products are manufactured by the supporting organization, packaged, and supplied to the managing pharmacist of the trial institution. Labeling of investigational medicinal products should be done in accordance with the regulation on drug manufacturing and quality control of Republic of Korea as follows:

1) Indication that it is “for clinical trial use.”
2) Name of investigational medicinal product
3) Batch or code number to identify the contents and packaging operation
4) Sponsor's name, address, and telephone number
5) Expiration date
6) Storage conditions
7) Reference codes to identify the trial
8) Provision and Storage

Investigational medicinal products are supplied by the supporting organization to clinical trial sites. The managing pharmacist of trial institutions will check the quantity and condition of the investigational medicinal products received. The managing pharmacist should handle and store the investigational medicinal products safely and appropriately and should keep the products in a safe place designated by the principal investigator where it is accessible only to the designated person. Administered investigational medicinal products should all be recorded in detail, including quantity and date of administration. Returned investigational medicinal products should all be recorded in detail, including quantity and date of return.

9. Study Subjects

(1) Inclusion Criteria
Eligible subjects will be considered for inclusion in this study if they meet all of the following criteria:

1) Men and women ≥ 19 years of age.
2) Diagnosed with aggressive B cell non-Hodgkin lymphoma;
   - Diffuse large B cell lymphoma (Both GCB and non-GCB): GCB type should not be more than 40% (N=26) of whole study population (Would limit number of GCB patients by maximum of 26)
   - Primary mediastinal B cell lymphoma
   - Transformed follicular lymphoma
   - Small lymphocytic lymphoma with Richter transformation
3) Failed previous treatments and last dose administered must be more than 2-weeks ahead from enrollment
   - Should have received anti-CD20 based chemotherapy previously
   - Failed to at least two lines of therapy if patient is candidate for autologous stem cell transplantation
   - Failed frontline therapy if patient is ineligible for autologous stem cell transplantation
4) Eastern Cooperative Oncology Group (ECOG) performance status of 0, 1 or 2
5) Woman of childbearing potential (WOCBP) who are sexually active must have 2 negative urine hCG test prior to first dose, then every week for the first month of

study period, then every 4 weeks afterwards during treatment period if menses are regular or every 2 weeks if menses are irregular. Urine hCG test must be done 4 weeks after the last dose of acalabrutinib, lenalidomide and rituximab. WOCBP must use 2 methods including at least 1 highly effective method of contraception for 4 weeks prior to first dose, during treatment period and for 4 weeks after the last dose of acalabrutinib, lenalidomide and for 12 months after the last dose of rituximab. Men who are sexually active must use condoms during treatment period and 4 weeks after the last dose of any study drug.

6) Willing and able to participate in all required evaluations and procedures in this study protocol including swallowing capsules without difficulty.
7) Ability to understand the purpose and risks of the study and provide signed and dated informed consent and authorization to use protected health information

(2) Exclusion Criteria

Subjects will be ineligible for this study if they meet any of the following criteria:

1) Diagnosed with mantle cell lymphoma
2) Previously treated with more than four lines of chemotherapy (Consolidative autologous stem cell transplantation is deemed as the same line therapy)
3) Previously treated with allogeneic hematopoietic stem cell transplantation within 6 months
4) GVHD requiring treatment
5) Patient who cannot take drug per oral
6) Known resistance to both BTK inhibitor and lenalidomide (Progression free survival to both BTK inhibitor and lenalidomide < 6 months)
7) Known resistant mutation to BTK inhibitor (BTKC481S and PLGCR665W)
8) Prior malignancy (or any other malignancy requiring active treatment), except for adequately treated basal cell or squamous cell skin cancer, in situ cervical cancer, or other cancer from which the subject has been disease free for ≥ 5 years or which will not limit survival to < 5 years.
9) Clinically significant cardiovascular disease such as uncontrolled or symptomatic arrhythmias, congestive heart failure, or myocardial infarction within 6 months of screening, or any Class 3 or 4 cardiac disease as defined by the New York Heart Association Functional Classification. Subjects with controlled, asymptomatic atrial fibrillation and atrial flutter during screening can enroll on study.
10) Malabsorption syndrome, disease significantly affecting gastrointestinal function, or resection of the stomach or small bowel that is likely to affect absorption, symptomatic inflammatory bowel disease, partial or complete bowel obstruction, or gastric restrictions and bariatric surgery, such as gastric bypass.
11) Known history of infection with HIV or any uncontrolled active systemic infection (eg, bacterial, viral or fungal).
12) Known history of drug-specific hypersensitivity or anaphylaxis to study drug (including active product or excipient components).
13) Active bleeding or history of bleeding diathesis (eg, hemophilia or von Willebrand disease).
14) Uncontrolled AIHA (autoimmune hemolytic anemia) or ITP (idiopathic thrombocytopenic purpura).
15) Requires treatment with a strong cytochrome P450 3A4 (CYP3A4) inhibitor/inducer.

16) Requires or receiving anticoagulation with warfarin or equivalent vitamin K antagonists (eg, phenprocoumon) within 7 days of first dose of study drug.
17) Prothrombin time (PT)/INR or aPTT (in the absence of lupus anticoagulant) >2x ULN.
18) Requires treatment with proton pump inhibitors (eg, omeprazole, esomeprazole, lansoprazole, dexlansoprazole, rabeprazole, or pantoprazole). Subjects receiving proton pump inhibitors who switch to H2-receptor antagonists or antacids are eligible for enrollment to this study.
19) History of significant cerebrovascular disease/event, including stroke or intracranial hemorrhage, within 6 months before the first dose of study drug.
20) Major surgical procedure within 28 days of first dose of study drug. Note: If a subject had major surgery, they must have recovered adequately from any toxicity and/or complications from the intervention before the first dose of study drug.
21) Hepatitis B or C serologic status: subjects who are hepatitis B core antibody (anti-HBc) positive and who are surface antigen negative will need to have a negative polymerase chain reaction (PCR). Those who are hepatitis B surface antigen (HbsAg) positive or hepatitis B PCR positive will be excluded. Those who are positive only for anti-HBc and negative for HbsAg and hepatitis B PCR need to receive adequate antiviral prophylaxis for hepatitis B during the study period. Subjects who are hepatitis C antibody positive will need to have a negative PCR result. Those who are hepatitis C PCR positive will be excluded.
22) Active tuberculosis (history of exposure or history of positive tuberculin test; plus presence of clinical symptoms, physical or radiographic findings). Subjects with latent tuberculosis infection who are deemed to require treatment by the investigator are not eligible.
23) Uncontrolled active infection as determined by the investigator.
24) WBC < 3,000 /μL, ANC < 1,000 /μL, Platelets < 75,000 /μL, or Hemoglobin < 9.0 g/dL. Correction with transfusion within 2 weeks is not allowed.
25) Total bilirubin > 2 x ULN, or AST, ALT > 3 x ULN
26) Cr > 1.5 x ULN or CLcr < 30 mL/min/1.73m2
27) Breastfeeding or pregnant.
28) Concurrent participation in another therapeutic clinical trial.

(3) Rationale for Sample Size
In the previous study for DLBCL patients who failed previous treatment regimen containing rituximab, overall response rate with salvage chemotherapy was around 27%.[4] Considering the efficacy of combination regimen with rituximab and lenalidomide and monotherapy with ibrutinib, we assumed ORR for our study wound reach around 45%. Under this assumptions, number of patients to draw conclusion with alpha error rate of 5% and power of 80% is 60. Considering dropout rate of 10%, total of 66 patients are necessary.

10. Intervention Protocol
(1) Intervention

This study is prospective, open-label, single arm phase II study to assess efficacy and safety of acalabrutinib, rituximab, lenalidomide (R2A) regimen on R/R B-cell NHL. The subjects are outpatients or inpatients who satisfy the inclusion and exclusion criteria and must agree and provide informed consent to participate after they were explained with the purpose and risks of

the study. All patients who were included in the study must have history taking, blood samples, chest X-ray, electrocardiogram, beta-hCG (in case of female subject of childbearing age) evaluated within 2 weeks and chest CT, abdomen/pelvis CT, and FDG PET/CT within 4 weeks. Additional examination with bone marrow biopsy, CT, MRI, endoscopes may be done if considered necessary by investigators. After the subject has signed and dated the Informed Consent Form (ICF), all screening procedures have been completed, and eligibility has been confirmed, the subject can be enrolled into the study.

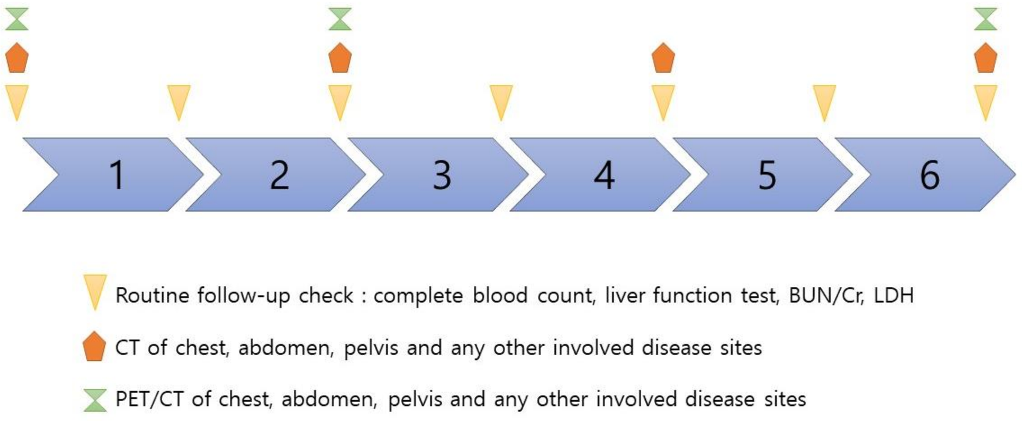

Before administration of investigational medicinal products, all patients must undergo pretreatment biopsy of the tumor. The pretreatment biopsy must include at least 2 core biopsy samples of the tumor.

Rituximab 375 mg/m2 will be administered intravenously on day 1. Lenalidomide 20 mg will be administered orally once daily from day 1 to day 21 of each cycle. Acalabrutinib 100 mg will be administered orally twice daily from day 1 to day 28. Day 1 to day 28 is considered one cycle, and cycles will be repeated every 4-weeks for a total of 6 cycles.

Duration of treatment is until completion of 6 cycles of R2A. Those who respond to R2A regimen, defined as best response being a PR or CR, maintenance therapy with acalabrutinib 100 mg BID monotherapy for a period of 1 year will be offered.

For safety concern, we are going to run first 3 patients as safety cohort and start with reduced dose of lenalidomide. To check safety, the first 3 patients would start with lenalidomide 15 mg once daily. Dosing of lenalidomide on subsequent cycle (at the second cycle) would be 20 mg once daily if there is no more than grade 2 hematologic toxicity attributable to study drug at D28, and 15 mg once daily if there was more than grade 2 hematologic toxicity attributable to study drug but recovered to less than Grade 2 at D35. If 2 out of 3 patients tolerate lenalidomide 15 mg once daily regimen, then the 4th patient and beyond will receive lenalidomide 20 mg once daily initially. If 2 or more patients in the safety cohort failed to tolerate lenalidomide 15 mg once daily regimen, then the 4th patient and beyond will receive lenalidomide 15 mg once daily initially. If any non-hematologic toxicity of grade 3 or more occurs in the safety cohort, the 4th patient and beyond will receive lenalidomide 15 mg once daily initially.

On-treatment CT scans with contrast (unless contraindicated) of the chest, abdomen, pelvis and any other disease sites will be done for tumor assessments at Week 8, 16, 24 (± 7 days),

then every 3 months (± 14 days) thereafter, or more frequently at investigator discretion. If contrast is contraindicated, contrast enhanced CT may be substituted with non-contrast CT scan or MRI of the chest, abdomen, pelvis and any other disease sites as per investigator's decision. On-treatment PET/CT scan of the chest, abdomen, pelvis and any other disease sites will be done for tumor assessments at Week 8 (± 7 days). PET/CT scan can be done at Week 24 (± 7 days) according to investigators decisions. At all other visits, tumor assessments will be done by physical exam and laboratory results. Response assessments will be evaluated based on Lugano Criteria

(2) Comparator

This study is a single-arm phase II clinical trial. Therefore, there is no comparator group in this study.

(3) Administration of investigational medicinal products

1) Administration schedule

For days where subjects take both acalabrutinib and lenalidomide, acalabrutinib and lenalidomide can be taken together at the same time. Rituximab can be administered regardless of timing of administration of acalabrutinib and lenalidomide.

2) Acalabrutinib

Acalabrutinib capsule is administered BID and taken orally approximately every 12 hours.

The capsules should be swallowed intact with water. Subjects should not attempt to open capsules or dissolve them in water. Acalabrutinib can be taken with or without food.

If a dose is missed, it can be taken up to 3 hours after the scheduled time with a return to the normal schedule with the next dose. If it has been > 3 hours, the dose should not be taken and the subject should take the next dose at the scheduled time. The missed dose will not be made up and must be returned to the site at the next scheduled visit.

3) Rituximab

Rituximab vial is administered as an intravenous infusion. Premedication with acetaminophen and an antihistamine before each infusion of rituximab is required unless contraindicated. Dexamethasone 40 mg should be administered prior to each infusion. Vital signs (blood pressure, pulse, oxygen saturation and body temperature) should be checked right before initiation of every rituximab infusion and every time before increment of rituximab infusion rate.

For first infusion, initiate infusion at a rate of 50 mg/hr. In the absence of infusion toxicity, increase infusion rate by 50 mg/hr increments every 30 minutes, to a maximum of 400 mg/hr.

For subsequent infusion, initiate infusion at a rate of 100 mg/hr. In the absence of infusion toxicity, increase rate by 100 mg/hr increments at 30-minute intervals, to a maximum of 400 mg/hr.

Interrupt the infusion or slow the infusion rate for infusion-related reactions. Continue the infusion at one-half the previous rate upon improvement of symptoms. In case of severe infusion reaction or hypersensitivity, desensitization protocol may be applied as per investigator's decision.

4) Lenalidomide

Lenalidomide should be taken orally at about the same time each day, either with or without food. Lenalidomide capsules should be swallowed whole with water. The capsules should not be opened, broken, or chewed.

5) Compliance assurance

For each subject, acalabrutinib and lenalidomide compliance will be described in terms of the proportion of study drug actually taken as assessed by pill count. All subjects will be instructed to return all remaining medications each cycle, and not to share the study drugs with any other person. The returned medications must not be given back to the subject.

6) Dosing delays and modifications

Subjects should be followed closely for AEs or laboratory abnormalities that might indicate treatment-emergent AEs related to investigational medicinal products. If a subject experiences a treatment-related toxicity or other intolerable AE during the course of therapy, then the offending medication should be withheld, as necessary, until the AE resolves or stabilizes to an acceptable degree. For hematologic toxicities including thrombocytopenia and neutropenia, dose modification for lenalidomide should be considered first. After first dose modifications for lenalidomide due to the hematologic toxicities have been done and still the hematologic toxicities persist, dose modifications for acalabrutinib should be considered.

7) Acalabrutinib dose modifications

Dose modifications for the following treatment-emergent AEs that the investigator considers to be causally related to acalabrutinib are provided in Table 3-1:

- Grade 4 neutropenia (< 500/μL) for > 7 days (neutrophil growth factors are permitted per American Society of Clinical Oncology (ASCO) guidelines[24] and use must be recorded on the case report form [CRF]).

- Grade 3 thrombocytopenia in presence of significant bleeding.

- Grade 4 thrombocytopenia.

- Grade 3 or 4 nausea, vomiting, or diarrhea, if persistent despite optimal antiemetic and/or anti-diarrheal therapy.
- Any other Grade 4 toxicity or unmanageable Grade 3 toxicity.

Table: Drug Modification Actions for Acalabrutinib

Occurrence	Action
1st – 2nd	Hold acalabrutinib until recovery to Grade ≤ 1 or baseline; may restart at original dose level
3rd	Hold acalabrutinib until recovery to Grade ≤ 1 or baseline; restart at one dose level lower (100 mg QD)
4th	Discontinue acalabrutinib

As appropriate, certain laboratory abnormalities may warrant more frequent monitoring (eg, once per week) until abnormalities have recovered to Grade ≤ 1. If acalabrutinib is reduced for apparent treatment-related toxicity, the dose need not be re-escalated, even if there is minimal or no toxicity with the reduced dose. However, if the subject tolerates a reduced dose of acalabrutinib for ≥ 4 weeks then the dose may be increased to the next higher dose level, at the discretion of the investigator. Such re-escalation may be particularly warranted if further evaluation reveals that the AE that led to the dose reduction was not treatment-related. The maximum dose of acalabrutinib is 100 mg BID.

Treatment with acalabrutinib should be withheld for any unmanageable, potentially study drug-related toxicity that is Grade ≥ 3 in severity. Any other clinically important events where dose delays may be considered appropriate must be discussed with the Principal Investigator.

8) Rituximab dose modification

There are no dose modification protocols for rituximab. Treatment with rituximab should be withheld for any unmanageable, potentially study drug-related toxicity that is Grade ≥ 3 in severity.

For a subject who experiences Grade ≤ 3 infusion reactions, desensitization protocols may be applied per each institution’s policy. Rituximab administration is contraindicated for the subject who experienced Grade 4 infusion reactions caused by rituximab.

9) Lenalidomide dose modification

Lenalidomide is associated with increased incidence of deep vein thrombosis and pulmonary embolism. Thromboprophylaxis with either aspirin or low dose heparin is recommended per investigator's decision. However, as acalabrutinib is associated with an increased risk of bleeding, caution should be exercised.

Dose modifications for hematologic toxicities is summarized in the table below.

- Grade 4 neutropenia (< 500/µL) for > 7 days (neutrophil growth factors are permitted per American Society of Clinical Oncology (ASCO) guidelines[24] and use must be recorded on the case report form).

- Grade 3 thrombocytopenia in presence of significant bleeding.

- Grade 4 thrombocytopenia.

Table: Drug Modification Actions for Lenalidomide (Hematologic toxicity)

Occurrence	Action
1st	Interrupt lenalidomide treatment and follow CBC weekly until recovery to Grade ≤ 1 or baseline; may restart at original dose level
2nd	Interrupt lenalidomide treatment and follow CBC weekly until recovery to Grade ≤ 1 or baseline; restart at 5 mg less than the previous dose. Dose below 5 mg daily is not allowed, and consider discontinuation

Dose adjustments for renal impairment are shown in table 3-3. If a subject experiences decline of CLcr to less than 30 mL/min judged to be related to lenalidomide, hold the drug until the CLcr recovers to more than 30 mL/min. Then restart the drug at the physician's discretion at next lower dose level. If decreased CLcr < 30 ml/min that is judged to be related to any investigational medicinal product and persists for > 28 days, the subject must be withdrawn from study treatment.

Table: Dose Adjustment for Lenalidomide

Renal Function (Cockcroft-Gault)	Dose
CLcr 30 to 60 mL/min	10 mg once daily
CLcr < 30 mL/min (not requiring dialysis)	15 mg every other day
CLcr < 30 mL/min (requiring dialysis)	5 mg once daily. On dialysis days, administer the dose following dialysis

For other Grade 3/4 toxicities judged to be related to lenalidomide, hold drug and restart at the physician's discretion at next lower dose level when toxicity has resolved to ≤ Grade 2. Acalabrutinib and rituximab may be continued as long as the toxicity does not meet the criteria for delayed dosing.

10) Concomitant therapy

Standard supportive care medications are permitted as per institutional standards.

11) Prohibited concomitant therapy

Any chemotherapy, anticancer immunotherapy, corticosteroids (at dosages equivalent to prednisone > 20 mg/day for longer than 2 weeks) except for premedication of rituximab per study protocols, experimental therapy, or radiotherapy for treating R/R B-cell NHL are prohibited. Warfarin or equivalent vitamin K antagonists (eg, phenprocoumon) are prohibited.

The concomitant use of strong inhibitors/inducers of CYP3A4 should be avoided when possible. If a subject requires a strong CYP3A inhibitor while on study, monitor the subject closely for potential toxicities.

Strong Inhibitors of CYP3A	Strong Inducers of CYP3A
boceprevir	carbamazepine
clarithromycin	phenytoin
conivaptin	rifampin
indinavir	St John's wort
itraconazole	
ketoconazole	
lopinavir/ritonavir (combination drug)	
mibefradil	
nefazodone	
nelfinavir	
posaconazole	
ritonavir	
saquinavir	
telaprevir	

telithromycin	
voriconazole	

11. Study parameters

(1) Primary objective: Overall response rate by Lugano criteria

(2) Secondary objective: DoR, CR rate, PFS, OS, Treatment related AE(s), SAE, AEs leading to discontinuation of investigational medicinal products, quality of life assessments by EORTC-QLQ-C30, next generation sequencing study with pretreatment biopsy of tumor,

(3) Schedule of Assessments

Evaluation	Visit										
	Screening period	Treatment period (28-day cycle)								Follow-up period	
		1st		2nd	3rd	4th	5th	6th	Treatment termination	SFU	Survival
Days	-28 ~ -1	1	15	1	1	1	1	1	Within 10 days of the last dose of study drug	30 days after last dose of drug	Every 12 weeks
Informed consent	V										
Medical history	V										
Disease History	V										
Adverse events		V	V	V	V	V	V	V	V	V	V
Concomitant medications and therapy	V	V	V	V	V	V	V	V	V	V	
Confirmation of eligibility	V										
ECOG	V	V		V	V	V	V	V	V	V	
Physical examination	V	V	V	V	V	V	V	V	V	V	V
EORTC-QLQ-C30	V				V				V		
Height (at screening only) & Weight	V	V		V	V	V	V	V			

ECG	V										
Urine hCG test	V	V	V	V	V	V	V	V	V	V	
CBC	V	V	V	V	V	V	V	V	V	V	
Serum chemistry	V	V		V	V	V	V	V	V	V	
Hepatitis B and C testing	V										
Biomarker studies with blood sample	V				V		V		V		
Compliance measure		V	V	V	V	V	V	V	V		
Tumor biopsy	V										
Drug administration											
Acalabrutinib		V	V	V	V	V	V	V	V		
Rituximab		V		V	V	V	V	V			
Lenalidomide		V	V	V	V	V	V	V			
Tumor assessment											
CT	V				V		V		V		
PET/CT	V				V				V		

12. Adverse events

(1) Acalabrutinib

Specific adverse events of acalabrutinib that warrants the attention are followings.

1) Hemorrhage
2) Infection
3) Cytopenia
4) Secondary malignancies
5) Atrial fibrillation AND atrial flutter

(2) Rituximab

Specific adverse events of rituximab that warrants the attention are followings.

1) Infusion reactions
2) Severe Mucocutaneous Reactions
3) Hepatitis B Virus Reactivation
4) Progressive Multifocal Leukoencephalopathy
5) Tumor Lysis Syndrome
6) Infections
7) Cardiovascular Adverse Reactions
8) Renal Toxicity
9) Bowel Obstruction and Perforation
10) Decreased effects of Immunization
11) Embryo-Fetal Toxicity

(3) Lenalidomide

Specific adverse events of lenalidomide that warrants the attention are followings.

1) Embryo-Fetal Toxicity
2) Hematologic Toxicity
3) Venous and Arterial Thromboembolism
4) Second Primary Malignancies

5) Hepatotoxicity

6) Severe Cutaneous Reactions Including Hypersensitivity Reactions

7) Tumor Lysis Syndrome

8) Tumor Flare Reactions

9) Impaired Stem Cell Mobilization

13. Withdrawal and Removal of Subjects from Study Treatment

The investigator may withdraw any subject from study treatment, if, in the investigator's opinion, it is not in the subject's best interest to continue.

Any subject has the right to withdraw from the study at any time. In addition, subjects may be withdrawn from study treatment for the following reasons:

- Study treatment should be discontinued in the event of a toxicity lasting > 28 days, unless reviewed and approved by the investigator.
- Any subject who starts new chemotherapy or chemoimmunotherapy for the treatment of R/R B-cell NHL, becomes pregnant or breastfeeding, is significantly noncompliant, etc. should be withdrawn from study treatment.

Subjects who discontinue study therapy will continue to be followed on study for follow-up of safety and survival unless they withdraw consent for further follow-up. Thus, all subjects receiving ≥ 1 dose of study drug will be followed during the immediate post-therapy and long-term follow-up assessments unless the subject withdraws consent for such follow-up to be conducted.

Reasons for removal of a subject from the study are:

- Subject's withdrawal of consent from study
- Decision by principal investigator
- Subject lost to follow-up
- Death

14. Assessments of safety

Safety assessments will consist of monitoring and recording, AEs and SAEs; measurements of protocol-specified hematology, clinical chemistry, urinalysis, and other laboratory variables; measurement of protocol-specified vital signs; and other protocol-specified tests that are deemed critical to the safety evaluation of the study drug(s).

(1) Definition

1) Adverse Events

An AE is any unfavorable and unintended sign, symptom, or disease temporally associated with the use of an investigational (medicinal) product, regardless of attribution. This includes the following:

- AEs not previously observed in the subject that emerge during the protocol-specified AE reporting period.
- Pre-existing medical conditions (other than the condition being studied) judged by the investigator to have worsened in severity or frequency or changed in character during the protocol-specified AE reporting period.
- Abnormal laboratory values considered clinically significant by the investigator should be reported as an AE.

The following are NOT considered an AE:

- Pre-existing condition that has not worsened: A pre-existing condition (documented on the medical history CRF) is not considered an AE unless the severity, frequency, or character of the event worsens during the study period.
- Preplanned hospitalization: A hospitalization planned before signing the ICF is not considered an SAE, but rather a therapeutic intervention. However, if during the preplanned hospitalization an event occurs, which prolongs the hospitalization or meets any other SAE criteria, the event will be considered an SAE. Surgeries or interventions that were under consideration, but not performed before signing the ICF, will not be considered serious if they are performed after signing the ICF for a condition that has not changed from its baseline level. Elective hospitalizations for social reasons, solely for the administration of chemotherapy, or due to long travel distances are also not SAEs.
- Diagnostic testing and procedures: Testing and procedures should not be reported as AEs or SAEs, but rather the cause for the test or procedure should be reported. If a test or procedure is done to rule out a diagnosis, the sign or symptom leading to the test/procedure should be the event term, and the event term should only be updated to the diagnosis if/when the diagnosis is confirmed. Testing and procedures performed solely as screening measures (eg, routine screening mammography or colonoscopy) should not be reported as AEs or SAEs.
- Abnormal laboratory results that the investigator considers to not be clinically significant: Abnormal laboratory results are not AEs unless they are clinically significant. For example, a clinically significant laboratory result is one that requires treatment (for example a blood transfusion for low hemoglobin) or

requires a change in study drug (eg, lowering the dose or withholding study drug while the laboratory finding resolves or stabilizes).

- Progression of underlying malignancy: Progression of underlying malignancy will not be reported as an AE if it is clearly consistent with the suspected progression of the underlying cancer. Hospitalization due solely to the progression of underlying malignancy should NOT be reported as an SAE. Clinical symptoms of progression may be reported as AEs if the symptoms cannot be determined as exclusively due to the progression of the underlying malignancy, or if they do not fit the expected pattern of progression for the disease under study.

If there is any uncertainty about an AE being due only to the disease under study, it should be reported as an AE or SAE.

Symptomatic deterioration may occur in some subjects. Symptomatic deterioration is when progression is evident in the subject's clinical symptoms and the investigator may elect not to perform further disease assessments. If there is any uncertainty about an AE being due only to the disease under study, it should be reported as an AE or SAE.

2) Serious Adverse Event

An AE should be classified as an SAE if it meets any 1 of the following criteria:

- It results in death (ie, the AE actually causes or leads to death).
- It is life-threatening (ie, the AE, in the view of the investigator, places the subject at immediate risk of death. It does not include an AE that, had it occurred in a more severe form, might have caused death).
- It requires or prolongs inpatient hospitalization.
- It results in persistent or significant disability/incapacity (ie, the AE results in substantial disruption of the subject's ability to conduct normal life functions).
- It results in a congenital anomaly/birth defect in a neonate/infant born to a mother exposed to the investigational product.
- It is considered a significant medical event by the investigator based on medical judgment (eg, may jeopardize the subject or may require medical/surgical intervention to prevent 1 of the outcomes listed above).

3) Severity

Definitions found in the CTCAE version 5.0 or higher will be used for grading the severity (intensity) of AEs. The CTCAE displays Grades 1 through 5 with unique clinical descriptions of severity for each referenced AE. Should a subject

experience any AE not listed in the CTCAE, the following grading system should be used to assess severity:

- Grade 1 (Mild AE) – experiences which are usually transient, requiring no special treatment, and not interfering with the subject's daily activities
- Grade 2 (Moderate AE) – experiences which introduce some level of inconvenience or concern to the subject, and which may interfere with daily activities, but are usually ameliorated by simple therapeutic measures
- Grade 3 (Severe AE) – experiences which are unacceptable or intolerable, significantly interrupt the subject's usual daily activity, and require systemic drug therapy or other treatment
- Grade 4 (Life-threatening or disabling AE) – experiences which cause the subject to be in imminent danger of death
- Grade 5 (Death related to AE) – experiences which result in subject death

(2) Documenting and Reporting of Adverse and Serious Adverse Events

The investigator is responsible for ensuring that all AEs and SAEs that are observed or reported during the study are recorded on the CRF.

1) Adverse Event Reporting Period

The AE reporting period for this study begins when the subject receives the first dose of study drug and ends with the SFU visit which occurs 30 days after the last dose of investigational medicinal products. An exception to this reporting period is any AE occurring due to a protocol-defined screening procedure. If any SAE occurs beyond 30 days after the last dose of investigational medicinal products *AND* it is assessed by the investigator as related to investigational medicinal products, it must be reported as an SAE.

2) Assessment of Adverse Events

Investigators will assess the occurrence of AEs and SAEs at all subject evaluation timepoints during the study. All AEs and SAEs whether volunteered by the subject, discovered by study personnel during questioning, or detected through physical examination, or other means, will be recorded in the subject's medical record and on the AE CRF.

Each recorded AE or SAE will be described by its diagnostic term, duration (eg, start and end dates), severity, regulatory seriousness criteria, if applicable, suspected relationship to the study drug, and any actions taken. The relationship of AEs to the study drug will be assessed by means of the question: 'Is there a reasonable possibility that the event may have been caused by the study drug?' per FDA guidance on safety reporting requirements.[25]

(3) Pregnancy

The Sponsor should report all pregnancies and pregnancies in the partners of subjects to the IRB and Regulatory Authorities per institutional and/or regulatory guidelines. Investigators should report any occurrences to principal investigator within 24 hours.

Any uncomplicated pregnancy that occurs with the subject or with the partner of a treated subject during this study will be reported. All pregnancies and partner pregnancies that are identified during or after this study, wherein the estimated date of conception is determined to have occurred from the time of consent to 4 weeks after the last dose of acalabrutinib, lenalidomide and for 12 months after the last dose of rituximab will be reported, followed to conclusion, and the outcome reported.

Subjects should be instructed to immediately notify the investigator of any pregnancies. Any female subjects receiving study drug who become pregnant must immediately discontinue study drug. The investigator should counsel the subject, discussing any risks of continuing the pregnancy and any possible effects on the fetus.

(4) Expedited Reporting Requirements for Serious Adverse Events

The Sponsor should report all SAEs to the IRB and Regulatory Authorities per institutional and/or regulatory guidelines. Investigators should report any occurrences to principal investigator within 24 hours.

Whenever possible, SAEs should be reported by diagnosis term not as a constellation of symptoms. If the primary cause of death is disease progression, then death should not be reported as an AE/SAE. If the primary cause of death is not disease progression, then the death should be reported as a fatal/grade 5 SAE with the primary cause of death as the SAE term, as death is typically the outcome of the event, not the event itself. The primary cause of death on the autopsy report should be the term reported.

If study drug is discontinued because of an SAE, this information must be included in the SAE report.

(5) Type and Duration of Follow-up of Subjects after Adverse Events

All AEs and SAEs that are encountered during the protocol-specified AE reporting period should be followed to resolution, or until the investigator assesses the subject as stable, or the subject is lost to follow-up or withdraws consent.

(6) Study termination criteria

The study will be terminated with at any time after 10 patients are enrolled when the incidence of serious adverse events or death related to the study is 50% or more.

15. Endpoint Data Analysis

(1) Efficacy Endpoint(s)

The primary efficacy endpoint is ORR. Secondary endpoints include DOR, CR rate, PFS, OS.

(2) Safety Endpoint(s)

Analysis of safety parameters will be done with descriptive statistics. For each AE with at least 1 event, percentage of experienced subjects will be summarized.

(3) Analysis of Efficacy Parameters

1) Primary efficacy endpoint

Exact 95% CIs will be calculated for the objective response rate using Blythe-Still-Casella method.

2) Secondary Efficacy Endpoint

For subjects who achieve objective response, DOR is defined as the time from the first response to the time of disease progression or relapse or death due to any cause, whichever occurs first. Subjects who are still alive and free from progression at the time of data cutoff date, lost to follow-up, have discontinued study, or have initiated other non-protocol anti-tumor therapy will be censored at the last tumor assessment when subjects are progression-free. DOR will be estimated using Kaplan-Meier method. 95% CIs for median DOR will be computed using the formula proposed by Brookmeyer and Crowley.

CR is defined according to Lugano criteria, no evidence of disease at the time of tumor assessment.

PFS is defined as the time from first dose to documented disease progression, or death from any cause, whichever occurs first. Data for subjects who are still alive and free from progression at the time of data cutoff date, lost to follow-up, have discontinued the study, or have initiated other non-protocol anti-tumor therapy will be censored on last assessment (or, if no post-baseline tumor assessment, at the time of first dose plus 1 day). Duration of PFS will be estimated using Kaplan-Meier methodology. 95% CIs for median duration of PFS will be computed using the formula proposed by Brookmeyer and Crowley.

OS is defined as the time from first dose to death from any cause. Data for subjects who are still alive at the time of data cutoff date, lost to follow-up, have discontinued the study (or, if no post-baseline assessment, at the time of first dose plus 1 day). Duration of OS will be estimated using Kaplan-Meier methodology. 95% CIs for median duration of OS will be computed using the formula proposed by Brookmeyer and Crowley.

16. Reference

1. Dotan E, Aggarwal C, Smith MR. Impact of Rituximab (Rituxan) on the Treatment of B-Cell Non-Hodgkin's Lymphoma. *P&T* **35**, Table 3 (2010)

2. Bertrand Coiffier, M.D., Eric Lepage, M.D., Ph.D., Josette Brière, M.D., Raoul Herbrecht, M.D., Hervé Tilly, M.D., Reda Bouabdallah, M.D., Pierre Morel, M.D.,

Eric Van Den Neste, M.D., Gilles Salles, M.D., Ph.D., Philippe Gaulard, M.D., Felix Reyes, M.D., MD e. al. CHOP Chemotherapy plus Rituximab Compared with CHOP Alone in Elderly Patients with Diffuse Large-B-Cell Lymphoma. *N Engl J Med*. **346**, 235-242 (2002)

3. Friedberg JW. Relapsed/refractory diffuse large B-cell lymphoma. *Hematol / Am Soc Hematol Educ Progr*. **2011**, 498–505 (2011).

4. Hitz F, Connors JM, Gascoyne RD, Hoskins P, Moccia A, Savage KJ, et al. Outcome of patients with primary refractory diffuse large B cell lymphoma after R-CHOP treatment. *Ann Hematol*. **94**, 1839–43 (2015).

5. Zelenetz AD, Gordon LI, Abramson JS, Advani RH. NCCN Guidelines Version 4.2018; B-Cell Lymphomas. National Comprehensive Cancer Network. *National Comprehensive Cancer Network* (2018).

6. Seshadri T, Kuruvilla J, Crump M, Keating A. Salvage Therapy for Relapsed/Refractory Diffuse Large B Cell Lymphoma. *Biol Blood Marrow Transplant*. **14**, 259–67 (2008).

7. Morrison VA, Hamlin P, Soubeyran P, Stauder R, Wadhwa P, Aapro M, et al. Approach to therapy of diffuse large B-cell lymphoma in the elderly: The International society of geriatric oncology (SIOG) expert position commentary. *Ann Oncol*. **26**, 1058–68 (2015).

8. Wiernik PH, Lossos IS, Tuscano JM, Justice G, Vose JM, Cole CE, et al. Lenalidomide monotherapy in relapsed or refractory aggressive non-Hodgkin’s lymphoma. *J Clin Oncol*. **26**, 4952–7 (2008).

9. Ruan J, Martin P, Shah B, Schuster SJ, Smith SM, Furman RR, et al. Lenalidomide plus Rituximab as Initial Treatment for Mantle-Cell Lymphoma. *N Engl J Med*. **373**, 1835–44 (2015).

10. Zinzani PL, Pellegrini C, Argnani L, Broccoli A. Prolonged disease-free survival in elderly relapsed diffuse large B-cell lymphoma patients treated with lenalidomide plus rituximab. *Haematologica* **101**, e385–6 (2016).

11. Zaja F, Ferrero S, Caterina Stelitano, Ferrari A, Salvi F, Arcari A, et al. Second-line rituximab, lenalidomide, and bendamustine in mantle cell lymphoma: a phase II clinical trial of the Fondazione Italiana Linfomi. *Haematologica*. **102**, e203–6 (2017).

12. Tuscano JM, Dutia M, Chee K, Brunson A, Reed-Pease C, Abedi M, et al. Lenalidomide plus rituximab can produce durable clinical responses in patients with relapsed or refractory, indolent non-Hodgkin lymphoma. *Br J Haematol*. **165**, 375–81 (2014).

13. Khan WN. Regulation of B lymphocyte development and activation by Bruton’s tyrosine kinase. *Immunol Res*. **23**, 147–56 (2001).

14. Mohamed AJ, Yu L, Bäckesjö CM, Vargas L, Faryal R, Aints A, et al. Bruton’s tyrosine kinase (Btk): Function, regulation, and transformation with special emphasis

on the PH domain. *Immunol Rev*. **228**, 58–73 (2009).

15. Bradshaw JM. The Src, Syk, and Tec family kinases: Distinct types of molecular switches. *Cell Signal*. **22**, 1175–84 (2010).

16. Soucek L, Buggy JJ, Kortlever R, Adimoolam S, Monclús HA, Allende MTS, et al. Modeling Pharmacological Inhibition of Mast Cell Degranulation as a Therapy for Insulinoma. *Neoplasia* **13**, 1093-IN43 (2011).

17. Ponader S, Chen S, Buggy JJ, Balakrishnan K, Gandhi V, Wierda G, et al. The Bruton tyrosine kinase inhibitor PCI-32765 thwarts chronic lymphocytic leukemia cell survival and tissue homing in vitro and in vivo. *Blood*. **119**, 1182-9 (2012).

18. De Rooij MFM, Kuil A, Geest CR, Eldering E, Chang BY, Buggy JJ, et al. The clinically active BTK inhibitor PCI-32765 targets B-cell receptor- and chemokine-controlled adhesion and migration in chronic lymphocytic leukemia. *Blood*. **119**, 2590–4 (2012).

19. Wilson WH, Young RM, Schmitz R, Yang Y, Pittaluga S, Wright G, et al. Targeting B cell receptor signaling with ibrutinib in diffuse large B cell lymphoma. *Nat Med*. **21**, 922–6 (2015).

20. Wang ML, Rule S, Martin P, Goy A, Auer R, Kahl BS, et al. Targeting BTK with Ibrutinib in Relapsed or Refractory Mantle-Cell Lymphoma. *N Engl J Med*. **369**, 507–16 (2013).

21. Rushworth SA, Bowles KM, Barrera LN, Murray MY, Zaitseva L, MacEwan DJ. BTK inhibitor ibrutinib is cytotoxic to myeloma and potently enhances bortezomib and lenalidomide activities through NF-κB. *Cell Signal*. **25**, 106–12 (2013).

22. Jerkeman M, Eskelund CW, Hutchings M, Räty R, Wader KF, Laurell A, et al. Ibrutinib, lenalidomide, and rituximab in relapsed or refractory mantle cell lymphoma (PHILEMON): a multicentre, open-label, single-arm, phase 2 trial. *Lancet Haematol*. **5**, e109–16 (2018).

23. Schmitz R, Wright GW, Huang DW, Johnson CA, Phelan JD, Wang JQ, et al. Genetics and Pathogenesis of Diffuse Large B-Cell Lymphoma. *N Engl J Med*. **378**, 1396–407 (2018).

24. Smith TJ, Khatcheressian J, Lyman GH, et al. 2006 Update of recommendations for the use of white blood cell growth factors: An evidence-based clinical practice guideline. *J Clin Oncol*. **24**, 3187-3205 (2006).

25. FDA. Guidance for Industry and Investigators: Safety Reporting Requirements for INDs and BA/BE Studies. FDA Guidance for Industry.
